# Recombinant Adenovirus siRNA Knocking Down the Ndufs4 Gene Alleviates Myocardial Apoptosis Induced by Oxidative Stress Injury

**DOI:** 10.1155/2023/8141129

**Published:** 2023-01-27

**Authors:** Beibei Wang, Jinsheng Zhang, Aijun Xu

**Affiliations:** ^1^Department of Anesthesiology, Tongji Hospital of Tongji Medical College of Huazhong University of Science and Technology, Wuhan, China; ^2^Department of Anesthesiology, Huashan Hospital of Fudan University, Shanghai, China; ^3^Third Affiliated Hospital of Henan University of Traditional Chinese Medicine, Zhengzhou, Henan, China

## Abstract

Oxidative stress results in myocardial cell apoptosis and even life-threatening heart failure in myocardial ischemia-reperfusion injury. Specific blocking of the complex I could reduce cell apoptosis. Ndufs4 is a nuclear-encoded subunit of the mitochondrial complex I and participates in the electron transport chain. In this study, we designed and synthesized siRNA sequences knocking down the rat Ndufs4 gene, constructed recombinant adenovirus Ndufs4 siRNA (Ad-Ndufs4 siRNA), and primarily verified the role of Ndufs4 in oxidative stress injury. The results showed that the adenovirus infection rate was about 90%, and Ndufs4 mRNA and protein were decreased by 76.7% and 64.9%, respectively. Furthermore, the flow cytometry assay indicated that the cell apoptosis rate of the Ndufs4 siRNA group was significantly decreased as compared with the H_2_O_2_-treated group. In conclusion, we successfully constructed Ndufs4 siRNA recombinant adenovirus; furthermore, the downexpression of the Ndufs4 gene may alleviate H_2_O_2_-induced H9c2 cell apoptosis.

## 1. Introduction

Over the past decades, there has been an increase in infarct cardiomyopathy all over the world, the effective disposal of which is rapid reperfusion. However, reestablishing blood supply could paradoxically exacerbate tissue injury, which is called ischemia/reperfusion (I/R) injury [1, 2]. I/R injury is a complex pathophysiological process, which is often linked to myocardial cell dysfunction, apoptosis, and even cell death [3, 4]. In I/R injury processes, oxidative stress plays a pivotal role [5–7]. Current research studies indicate that oxidative stress promotes cardiomyocyte necrosis and apoptosis through mitochondria, endoplasmic reticulum, or death receptors [7–12]. Previous research has established that oxidative stress damage is mainly related to reactive oxygen species (ROS) overproduction [13, 14]. The major source of ROS is thought to be the mitochondrial complex I during oxidative stress injury [15]. Moreover, a significant number of studies have shown that specific blockage of the complex I electron transport chain could protect the myocardium from oxidative stress injury through a variety of mechanisms [16–19]. However, the effective blocking site is still elusive.

Ndufs4, a nuclear-encoded subunit of the mitochondrial complex I, plays an important role in mitochondrial electron transport chain (ETC) [20–22]. A recent study suggested that heart-specific knockout of Ndufs4 may ameliorate I/R injury [23]. Therefore, the Ndufs4 subunit is a putatively potential site for blocking the ETC to protect myocardial cells from oxidative stress injury.

In recent years, gene therapy has emerged as a promising therapeutic avenue for the therapy for cancer [24] and even heart disease [25], with numerous studies reporting its positive effects on cardiac function in cultured cells or small animal models. Adenovirus is the most widely used vector for its merits of broad host range, rapid propagation period, high infection rate, and convenient storage [26]. Small interfering RNA (siRNA) is short double-stranded RNA that can induce specific mRNA degradation to reduce or silence the expression of target genes. To investigate whether blocking the ETC through the Ndufs4 subunit can protect cardiomyocytes during I/R injury, we applied the advantages of recombinant adenovirus and siRNA to specifically knock down the expression of the Ndufs4 gene.

In this study, to investigate the role of the Ndufs4 subunit in oxidative stress injury, we constructed recombinant adenovirus targeting the Ndufs4 gene (Ad-Ndufs4 siRNA) as well as the negative control sequence (Ad-U6-GFP). The knocking down efficiency of Ad-Ndufs4 siRNA in H9c2 cells was evaluated by real-time PCR and Western blot assays. Furthermore, oxidative stress injury was simulated with H_2_O_2_, and H9c2 cell apoptosis was tested by flow cytometry (FCM) assay.

## 2. Materials and Methods

### 2.1. Materials

The H9c2 cell line was gifted from the Division of Cardiothoracic and Vascular Surgery, Tongji Hospital, Tongji Medical College, Huazhong University of Science and Technology. Trizol and SYBR Master Mixture, and Taq polymerase were purchased from Takara (Japan). PCR reagent primer and dsDNA oligo were designed and compounded by GeneChem Company (Shanghai, China). QIAGEN Plasmid kit was purchased from QIAGEN (Germany). HEK293 and Luria–Bertani (LB) culture medium were purchased from ATCC (China). T4 DNA ligase and T4 DNA ligase buffer, Age I, EcoR I, Hpa I, and Xho I were obtained from NEB (USA), Dulbecco's modified eagle medium (DMEM), cell culture medium, and fetal bovine serum (FBS) were purchased from the GIBCO Company (Life Technologies, Shanghai, China). Annexin V-PE/7-AAD Dual Staining Cell Apoptosis Detection Kit was from BD biosciences (USA). The rabbit monoclonal antibody to Ndufs4 (ab139178) was obtained from Abcam (Cambridge, UK). Mouse anti-GAPDH antibody (KM9001) and goat antirabbit or goat antimouse secondary antibodies were obtained from Sungene Biotech (Tianjin, China).

### 2.2. Design and Synthesis of siRNA Sequences Targeting Rat Ndufs4 Gene

At first, based on the full-length CDS of the Ndufs4 gene (NM_001025146) and the RNA interference sequence design principles, the interference sequence was designed. It was CCAACATGGTTCTCACCTT, and the negative control sequence was TTCTCCGAACGTGTCACGT. Then, two single-stranded shRNA sequences were designed in line with these two siRNA sequences and were sent to GeneChem Company for synthesis.

### 2.3. Construction and Identification of Recombinant Shuttle Plasmid

The synthesized single-stranded DNA oligo and the primers were dissolved in an annealing buffer solution, followed by a water bath at 90°C for 15 minutes, and then naturally cooled to room temperature. Then, the shuttle plasmid GV120 was linearized by restriction enzymes Age I and EcoR I, and the linearized vector and DNA fragment were subjected to a ligation reaction in an appropriate buffer with T4 DNA ligase at 16°C overnight. Finally, ligation products were transformed into *E*. *coli* DH 5*α* competent cells using the thermal excitation method. The transformed cells grew in an LB medium containing 50 *μ*g/mL of ampicillin overnight at 37°C. Positive colonies were selected and propagated for colony sequencing to verify if the cloning was successful.

### 2.4. Packaging, Propagation, and Purification of Ad-Ndufs4 siRNA and Ad-U6-GFP

Before transfection, HEK293 cells were cultured for about 24 hours until their density reached 50%∼60% and were in good condition. Then, the DNA solution that contains the recombinant shuttle plasmid and auxiliary packaging plasmid was transferred to the medium of HEK293 cells, which was expected to be transfected with the help of Lipofectamine 2000. The cells were incubated in DMEM containing 10% FBS in 5% CO_2_, 37°C cell incubator for 10–12 days until the typical cytopathic effect (CPE) occurred in most cells, and 50% of the cells were detached from dishes. Cells were collected at low speed and resuspended in DMEM, repeatedly thawed and shaken at −80°C/37°C for 3 times, and centrifuged at 7000 g for 5 min at 4°C; thus, the virus supernatant was collected, that was the first-generationAd-Ndufs4 siRNA. Similarly, the Ad-U6-GFP was gained. HEK293 was repeatedly infected 3 times with the virus supernatant. The recombinant adenovirus crude extract was harvested and then purified by the Adeno-X™ Virus Purification Kit, then stored at −80°C.

### 2.5. Determination of Viral Titer of Ad-Ndufs4 siRNA

24 hours before the experiment, 100 *μ*L HEK293 cell suspension containing approximately 1 × 10^3^ cells was seeded to each well of a 96-well plate. Adenovirus stock solution 10 *μ*L was taken into a 990 *μ*L Ep tube to make a 1 : 100 dilution (10^−2^); then, we use this as a starting point and add 100 *μ*L of this dilution solution to a 900 *μ*L Ep tube for a 1 : 10 dilution (10^−3^) until diluted to 10^−13^. Then, 100 *μ*L diluted viral samples (10^−6^–10^−13^) were inoculated in the corresponding wells. Columns 11 and 12 were used as no-virus controls. The plates were incubated at 37°C, 5% CO_2_ for 10 days, then for each dilution, the number of wells positive for CPE was scored to calculate the virus titer (Spearman–Karber method).

### 2.6. H9c2 Cell Culture and the Ndufs4 Gene Knocking Down Efficiency Evaluation

H9c2 cells were cultivated in DMEM supplemented with 10% FBS and 100 U/mL penicillin and 100 *μ*g/mL streptomycin at 37°C, 5% CO_2_. They were divided into 3 groups: the control group (normally cultured H9c2 cells without any virus in the medium), the NC group (negative control group, add Ad-U6-GFP recombinant adenovirus), and the KD group (add Ad-Ndufs4 siRNA). According to pre-exposure experiments, the optimal infection condition for H9c2 cells to achieve 80% infection efficiency was normal medium, and the required multiplicity of infection(MOI) was 100 (the figures are not shown). Given this, H9c2 cells were seeded onto 6-well plates (about 5 × 10^4^ cells/well). Upon reaching 50%–60% confluence, H9c2 cells were infected with recombinant adenoviruses at MOI = 100. The efficiency of infection was evaluated by fluorescence microscopy, real-time PCR, and Western blot.

Real-time PCR was used to confirm the infection efficiency of recombinant adenoviruses. In brief, total RNA was extracted from the H9c2 cells using Trizol reagent according to the manufacturer's protocol, 0.5 *μ*g total RNA from each sample was used to generate cDNAs by the PrimerScript™ Master Mix Kit. Then, real-time PCR was performed to quantify the Ndufs4 expression level with SYBR Green PCR Master Mix. The forward and reverse primer sequences for Ndufs4 and GAPDH are listed in Table 1.

Western blot is another key indicator to verify the efficiency of recombinant adenovirus interference. Cells were homogenized in lysis buffer containing a mixture of protease and a phosphatase inhibitor cocktail and PMSF at the ratio of 100 : 1 : 1. Protein samples were separated on SDS-PAGE and transferred to PVDF membranes which then were blocked for 2 hours with 5% bovine serum albumin in TBST (0.1% Tween-20 in Tris-buffered saline) at room temperature, the membranes were then incubated overnight at 4°C with antibodies against Ndufs4 and GAPDH. Then, membranes were incubated for 2 hours with an appropriate secondary antibody. After being washed for 30 minutes, the bands were detected by the enhanced chemiluminescence method, and intensities were quantified using the Image J program.

### 2.7. Flow Cytometry Analysis of the Apoptosis of H9c2 Cells

H9c2 cells were seeded in 6-well plates (about 5 × 10^4^ cell/well). When the cell density reached approximately 50%, the H_2_O_2_ + NC and the H_2_O_2_ + siRNA groups were incubated with the Ad-U6-GFP and Ad-Ndufs4 siRNA, respectively. 24 h later, they were replaced with fresh medium and continued incubating for 48 h. And then, the medium of the H_2_O_2_ group (only add H_2_O_2_ without any adenovirus), the H_2_O_2_ + NC group, and the H_2_O_2_ + siRNA group were replaced with a medium containing 200 uM H_2_O_2_. Apoptosis was evaluated by flow cytometric analysis. In brief, the cells were washed twice with PBS and suspended in a 100 *μ*L binding buffer containing 5 *μ*L annexin V-fluorescein isothiocyanate (FITC) and 5 *μ*L propidium iodide (PI) in the dark at room temperature. 15 minutes later, the cell suspensions were immediately analyzed. Percentages of apoptotic cells were calculated as the ratio of annexin V-positive cells to the total cell population × 100.

### 2.8. Statistical Analysis

All data were analyzed using GraphPad Prism 5.0 software (GraphPad Software, San Diego, CA, USA). Shapiro–Wilk tests were performed to determine the normality of the data distribution, if data were normal distribution, then the one-way analysis of variance (ANOVA) was adopted and Tukey's post hoc test was applied for post hoc pairwise comparisons if the ANOVA test was significant. Data are expressed as mean ± SD. *P* < 0.05 was considered statistically significant.

## 3. Results

### 3.1. Construction of Recombinant Shuttle Plasmid Ndufs4 siRNA

**Figure 1:**
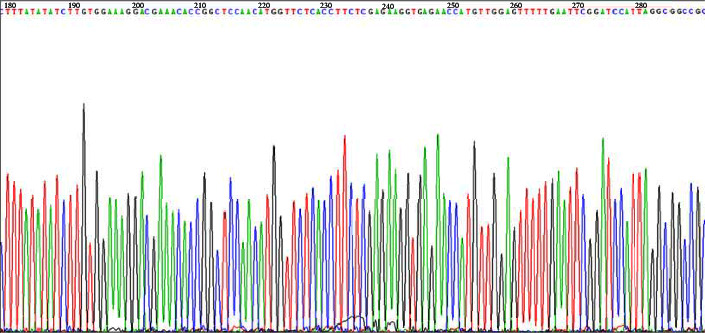
Analysis by Chromas sequencing software showed that the siRNA sequence was inserted correctly.

The interfering sequence oligonucleotide strand is listed in Table 2. After the linearized shuttle plasmid and DNA fragment were ligated by T4 DNA ligase, the ligation products were transformed into *E*. *coli* DH 5*α* competent cells. Analysis by Chromas sequencing software showed that the siRNA sequence was inserted correctly, and the sequencing results were exactly the same as the design sequence, which indicated that the construction of the shuttle plasmid was successful (Figure 1).

### 3.2. Packaging, Propagation, and Purification of Recombinant Adenovirus Ndufs4 siRNA

**Figure 2:**
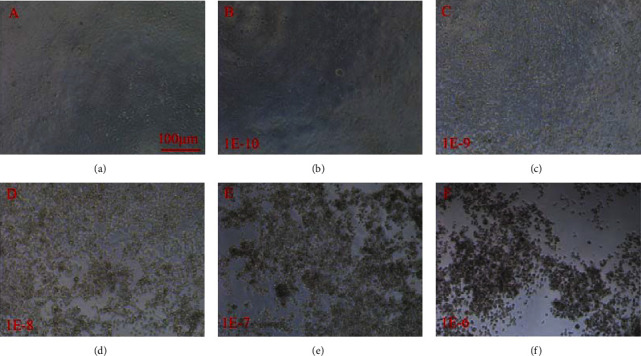
Morphological changes in HEK293 cells observed using the bright microscope. It showed that the morphology of the infected HEK293 was determined by the titers of Ad-Ndufs4 siRNA. The cells in the control group was normal, and the higher the virus titer, the greater frequency of CPE appearance.

The DNA solution containing the recombinant shuttle plasmid and auxiliary packaging plasmid were cocultured with HEK293 cells to package the Ndufs4 siRNA recombinant adenovirus. After 10 days, the typical cytopathic effect (CPE) occurred in most cells, and 50% of the cells detached from dishes. Adenovirus solutions were collected and then used to infect HK293 cells twice for higher-titer adenovirus, at last, 1 mL purified Ad-Ndufs4 siRNA and Ad-U6-GFP were harvested, respectively. The 50% tissue culture infective dose (TCID50) assay results revealed that the titer of Ndufs4 siRNA was 1 × 10^10^ PFU/mL (Figure 2).

### 3.3. The Knocking Down Efficiency of the Ndufs4 Gene by Ad-Ndufs4 siRNA

**Figure 3:**
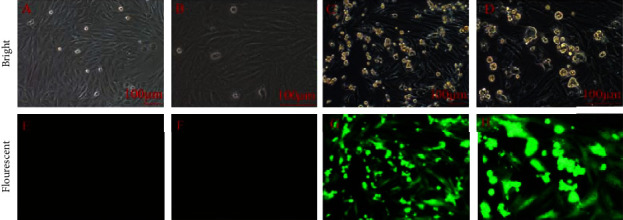
GFP expression in H9c2 cells. (a, e) H9c2 cells cultured under regular circumstances without virus were normal in the bright and fluorescent field, respectively (10x); (b, f) the same scene as (a) and (e) but under 20x field correspondingly; (c, f) H9c2 cells were incubated with recombinant adenoviruses at MOI = 100 in the bright and fluorescent field (10x); (d, g) the same scene as (c) and (f) but under 20x field (GFP: green fluorescent protein).

**Figure 4:**
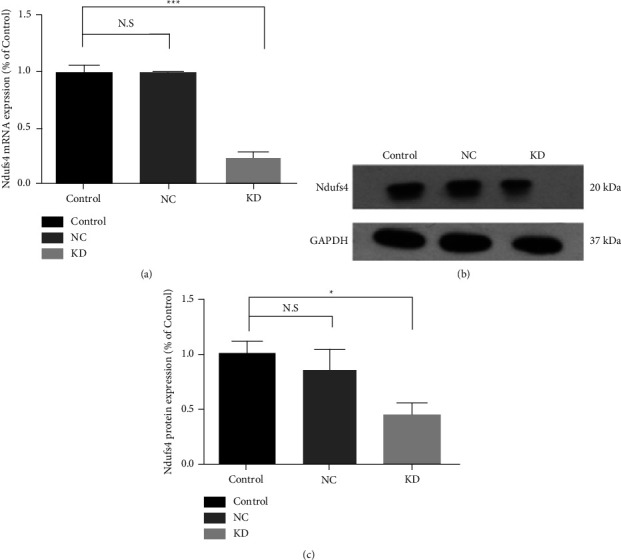
Ndufs4 gene was knocked down evidently. (a) The relative level of Ndufs4 mRNA in infected H9c2 cells was decreased by 76.7% versus the control group (*P* < 0.001). There was no significant difference between the control group and the NC group. (b, c) Western blot and quantitative evaluation of the expression of Ndufs4 gene in infected H9c2 cell lines. The protein content of Ndufs4 in the KD group decreased by 64.9% (*P* < 0.05) compared with the control group, and there was no significant difference between the control group and the NC group. Data were expressed as mean ± SD (*n* = 3). ^∗^*P* versus the control group; ^∗^*P* < 0.05 and ^∗^^∗^^∗^*P* < 0.001.

Transfected H9c2 expresses GFP, so the transfection efficiency was detected indirectly by fluorescence microscopy. After 72 hours of infection, the proportion of GFP-positive cells was over 90% (Figure 3). Real-time PCR and Western blot showed that compared with the control group, Ndufs4 mRNA level reduced by 76.7% in the KD group (*P* <  0.001) (Figure 4(a)), and the Ndufs4 protein level decreased by 64.9% (*P*  <  0.05) (Figures 4(b) and 4(c)).

### 3.4. Ndufs4 siRNA Prevented H_2_O_2_-Induced Apoptosis of H9c2 Cells

**Figure 5:**
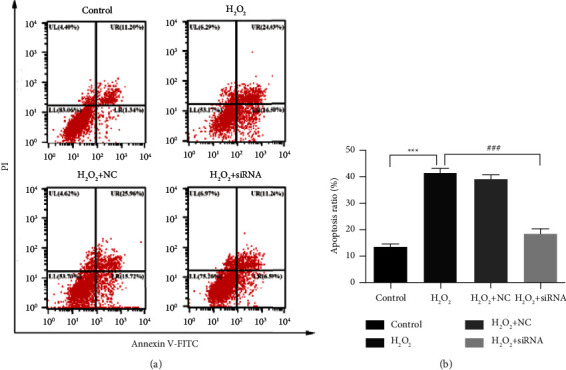
Knocking down Ndufs4 prevented H_2_O_2_-induced apoptosis in H9c2 cells. (a) Flow cytometry analyzed the H9c2 cell apoptosis ratio of each group after Annexin V-FITC/PI double staining. (b) The apoptosis ratio is shown in the graph. In the H_2_O_2_ group, cell apoptosis was significantly higher than that in the control group, but the cell apoptosis rate of the H_2_O_2_ + siRNA group was significantly lower than that of the H_2_O_2_ group (*P* < 0.001). Data were expressed as mean ± SD (*n* = 3). ^∗^*P* versus the control group, ^∗^^∗^^∗^*P* < 0.001; ^#^*P* versus the H_2_O_2_ group, ^###^*P* < 0.001; ^$^*P* versus the H_2_O_2_ + NC group, ^$$^*P* < 0.01.

In this experiment, flow cytometry was used to preliminarily test the apoptosis of H9c2 cells induced by H_2_O_2_. In the H_2_O_2_ group, the cell apoptosis rate was significantly higher than that in the control group, but the cell apoptosis rate of the H_2_O_2_ + siRNA group was significantly lower than that of the H_2_O_2_ group (*P* < 0.001) (Figure 5).

## 4. Discussion

In this study, we constructed recombinant adenovirus that expresses Ndufs4 siRNA and further reported that knocking down the Ndufs4 gene expression could protect cardiomyocytes from an oxidative stress injury. This is another new piece of evidence supporting that blockade of the complex I ETC ameliorates ischemia-reperfusion injury in cardiomyocytes, providing a new idea in cardiovascular disease gene therapy.

In this study, we successfully constructed Ndufs4 siRNA, the TCID50 was 1 × 10^10^ PFU/mL and transfected H9c2 was over 90% after 72 hours of infection. We also verified Ndufs4 siRNA effectiveness in H9c2 cells by Western blot and real-time PCR, the Ndufs4 siRNA reduced the expression of mRNA and protein of the Ndufs4 gene by 76.7% and 64.9%, respectively. In recent years, gene therapy has emerged as a promising therapeutic avenue for the therapy of some diseases, such as cancer or cardiovascular disease [25–28]. Owing to specific gene silencing, siRNA is expected to become an approach in treating some diseases [29].

Mitochondria is an important “energy factory” of eukaryotic cells. Complex I is the starting point of the respiratory chain, and it is also the largest complex, including 45 subunits [20, 30]. Ndufs4 is an important unit of complex I, in the one hand, it plays an important role in ECT in complex I, on the other hand, the Ndufs4 subunit is essential to normal growth and development for an individual. In humans, Ndufs4 mutation is causal for Leigh syndrome [31]. In this study, we knocked down the Ndufs4 by siRNA, we observed that compared with the control group, the growth and division of the cells were normal when the AAV MOI was under 150, but the cell morphology was slightly changed when the titer was higher (the figures are not shown), high titer virus itself may affect cell growth.

Mitochondria ETC plays a vital role in oxidative stress under either physiological or pathological conditions [32]. Since numerous experiments have demonstrated that blocking the ETC can reduce the oxidative stress injury of myocardial cells [18], such as amobarbital [16, 33]. In our study, flow cytometry analysis suggested that the reduction of Ndufs4 subunit expression can decrease the H9c2 apoptosis ratio from 40% to 18%, and mitigate H9c2 injury. A previous study examined that mesenchymal stem cells (MSCs) effectively lowered cellular ROS production in Ndufs4-deficient fibroblast cell lines, and CI protein expression and activity were not rescued by MSC treatment [34]. A recent study suggested that heart-specific knockout of Ndufs4 ameliorates ischemia-reperfusion injury [23], and another research even showed that low abundance of NDUFV2 and NDUFS4 subunits predicts mammalian longevity [22]. However, there is a study reported that Ndufs4 deficiency in mice rendered the myocardium more susceptible to I/R injury [35]. It indicated that the role of Ndusf4 deficiency in the cell, tissue, or whole body may be varied and complicated, and it deserve further research.

There are some limitations to this study. Although we found that cell apoptosis induced by H_2_O_2_ was decreased in the H_2_O_2_ + siRNA group, more experimental evidence should be adduced, and the underlying signaling pathway was not fully elucidated, which is the focus of our following study.

## 5. Conclusion

We successfully constructed the recombinant Ndufs4 siRNA vector which remarkably reduced Ndufs4 expression in H9c2 cells, the downexpression of the Ndufs4 gene may alleviate H_2_O_2_-induced H9c2 cell apoptosis.

## Figures and Tables

**Table 1:** Ndufs4 and GAPDH primer sequences for qRT-PCR.

Gene	Primer sequence
Ndufs4	F: 5ʹ-TGACAGGCGATACATCCATAT-3ʹ
	R: 5ʹ-GAAGTTCTCACAATCACCAGAT-3ʹ

GAPDH	F: 5ʹ-TTCAACGGCACAGTCAAGG-3ʹ
	R: 5ʹ-CTCAGCACCAGCATCACC-3ʹ

**Table 2:** The Ndufs4 interfering sequence oligonucleotide strand.

Ndufs4 RNAi	5ʹ	Stem	Loop	Stem	3ʹ
Forward	CCGG	ctCCAACATGGTTCTCACCTT	CTCGAG	AAGGTGAGAACCATGTTGGAG	TTTTTG
Reverse	AATTCAAAAA	ctCCAACATGGTTCTCACCTT	CTCGAG	AAGGTGAGAACCATGTTGGAG	

## Data Availability

The data used to support the findings of this study are included within the article and further inquiries can be directed to the corresponding author.
